# Role of Sport Vision in Performance: Systematic Review

**DOI:** 10.3390/jfmk9020092

**Published:** 2024-05-23

**Authors:** Andrea Buscemi, Flora Mondelli, Ilaria Biagini, Stella Gueli, Angela D’Agostino, Marinella Coco

**Affiliations:** 1 Italian Association of Orthoptists Assistants in Ophthalmology, 98039 Taormina, Italy; andreabuscemi@virgilio.it (A.B.); vicepresidenza@aiorao.it (F.M.); ilaria.biagini55@gmail.com (I.B.); 2 Italian Osteopathic Study Center, 95100 Catania, Italy; stellagueli58@gmail.com (S.G.); angela.dagostino.95@gmail.com (A.D.); 3 Social Cooperative Horus, 97100 Ragusa, Italy; 4 Department Neurofarba, University of Firenze, 50121 Firenze, Italy; 5 Department of Educational Sciences, University of Catania, 95100 Catania, Italy; 6 B.R.I.T. (Bio-Nanotech Research and Innovation Tower) Service Center, University of Catania, 95100 Catania, Italy

**Keywords:** sport vision, eyes, performance, cognition, orthoptic rehabilitation, osteopathic treatment

## Abstract

Sport Vision is a speciality of multidisciplinary interest aimed at improving the performance of the visual system to achieve benefits in practiced sports, as well as in daily life and in preventive care. The type of training practiced by the athlete, his or her physical condition, cognitive level, and level of fatigue condition affects the speed of the reaction time and, consequently, the speed of motor response. Specific orthoptic exercises, the use of technological devices, the recovery of static and dynamic postural stability by using unstable platforms and the dual-task paradigm can help to achieve the expected results. The aim of this systematic review of Sport Vision was to assess the overall existing literature on Sport Vision, paying particular attention to the effects of visual training and its application in different sports and in rehabilitation and preventive settings. We analysed published English language studies about the role of sport vision in athletic performance from 1950 to 2023. We searched through the Medline database. The PRISMA 2020 checklist was used to assess the transparency and reproducibility of this review. The enrolled papers were evaluated with the Jadad Scale, Amstar 2 Scale and Newcastle–Ottawa Scale. 25 (16 studies, 5 reviews, 2 comments, 1 editorial, 1 descriptive paper) out of 476 studies met the inclusion criteria. Due to the variability in the age of the samples, the different techniques, the treatments among the participants in the studies and the finding of non-evaluable articles, a meta-analysis was not conducted. The limitations of this review are the single database research, the studies analyzed contain a non-statistically representative sample size and the lack of a control group. There is no standardized test to measure performance. It was shown that the development of visual skills can benefit athletes in injury prevention, and can lead to improved sports performance and motor function at any age, acquiring adaptive motor behaviour even when the visual system is impaired, due to task repetition and familiarity of the gesture. We intended to identify a multidisciplinary approach and a manual treatment scheme to optimize the circuitry involved in sport vision in order to increase the results that are achieved, but further studies will be needed to this end.

## 1. Introduction

Sport Vision (SV) is a new speciality that has attracted particular interest over the past 20 years, especially in athletes looking for new ways to improve their visual skills to achieve a better performance on the court [1]. Nowadays, sport vision training is used to raise the level of play of athletes and outperform the competition. The agonists are distinguished by physical strength, speed, cognitive functions and endurance necessary to play, but only good visual processing and a high reaction speed are the elements that distinguish an excellent performance, and these can be improved with sport vision training in any athlete, regardless of the level of play [2]. The athlete with a better visual reaction speed will be able to see the game almost ‘in slow motion’, and therefore he will be able to react and make decisions in advance in fractions of seconds, positively influencing his performance [2].

The late Blanton Long Collier, a football coach of the University of Kentucky between 1954 and 1961 and the Cleveland Browns of the National Football League between 1963 and 1970, said that the eyes guide the body. He was one of the first coaches who recognized that not only sight is critical in performance, but also that its quality differs from player to player [3].

The term sight refers to the clarity of the image on the retina; on the other hand, the term vision has a deeper meaning which includes the mental process of what derives from what is observed, and it is the last result of the integrity of the visual circuit/path, visual efficiency and visual information processing [1]. The goal of the SV is to improve and preserve visual function to increase sports performance [1]. The term dates back to the 18th century, when the good eye began to be occluded in amblyopathic patients (lazy eye) but did not emerge until the end of the twentieth century when an optometrist began giving advice to a group of athletes in the United States [1]. In 1960, basketball players were subjected to eye examinations, which ten years later became part of the routine of services offered to the athletes. During the 1984 Olympics in Los Angeles, a series of visual tests were practiced on athletes, then repeated during the 2004 Olympics in Athens on a large scale [1]. The “European Academy of Sport Vision” (EASV) was created in 1988 in Rome, with the aim of training technicians specialized in this area. The EASV defines the SV as a multi-disciplinary functional approach aimed at leading the entire visual system to function at the peak of performance in relation to the activity practiced [4].

### 1.1. Sport Vision and Sports Skills

In the early 1980s, visual skills in sports emerged as relevant variables in athletic performance [4]. The difference in visual capacity development between athletes and non-athletes was evident, and the difference in performance between athletes with better visual abilities and those with poorer ones was considerable [4]. In fact, regardless of the sport practiced, planning the athletic gesture allows athletes to constantly stimulate the visual system achieving an improvement in their performance [4]. The main visual skills involved in sports are eye–limb coordination, static and dynamic acuity, peripheral vision, spatial focus, speed and distance of subjects and objects in the environment [4]. Among these, stereopsia, the perception of depth, is essential for the three-dimensional analysis of the environment [5].

### 1.2. The Interaction between Visual and Locomotor Systems

The visual and locomotor systems work together to support body movement, but little is known about the mechanism of interaction between them, which is particularly evident during locomotion on natural terrain or rough, uneven surfaces with obstacles (walking on smooth, unobstructed floors does not require vision to guide foot positioning) [6]. In fact, human locomotion through such environments requires precise coordination between the biomechanics of the step cycle and the eye movements, which collect the information necessary to guide and position the foot on the ground [6,7]. In 2018, Matthis demonstrated how walkers use their gaze to adapt to the pace to the path they are following in order to balance length, width, duration and the thrust and positioning phase of the foot to remain in a stable condition with a minimal energy expenditure [6].

It has been seen that ‘eye movements and locomotion are intimately linked in a way that reflects the integration of energy costs, environmental uncertainty and the momentary demands of the motor task’ [6]. It can be deduced, therefore, that the environment conditions the motor task and our decision-making [6,7]. Very little is known about the visual search process for identifying support points during locomotion along complex terrain. The research shows that humans have a limited time to identify a suitable place where to put their foot because the time between each step, which is about 400–500 ms, consents only 2–3 fixations per pass [7]. As a consequence, the research process needs to be efficient. Simple spatial features such as large patches of light indicating convex surfaces may be sufficient [7]. However, a distinctive feature of uneven terrain is the variation in depth in the walkable surface. These variations require the adjustment of the descending step so that the foot contacts the appropriate forces for that height. Therefore, the research process must identify a relatively smooth surface within the range of action and know its three-dimensional position [7]. Buckley et al. [8] found that subjects with an imposed visual impairment (occlusion or blurring of vision in one eye, induced to perform the test by blurring the input to one eye using a Bangerter Occlusion Foil) show a reduction in stereopsis and related tasks such as hand–eye coordination, slower walking, and raising their foot higher when climbing or overcoming an obstacle. Overall, individuals with reduced stereopsis have a more cautious gait, but the difference in performance in overcoming obstacles is evident only when the difficulty of the task increases, in this case, the height of the obstacle to be overcome [9]. This suggests an increased risk of falls while walking and indicates the important role of binocular vision in controlling foot placement. If compromised, there is a tendency to raise the margin of safety by increasing the height of the foot [9,10], but with the repetition of the task and the familiarity of the gesture the height of the foot is reduced, demonstrating how the motor behaviour is adaptive [10]. As far as the passage through a confined space, it was noted that individuals head first to the center of the path and then towards the center of the opening, but only the last two seconds before crossing they walk to where they were looking at [11]. This proves that only the final phase of the task is visually guided. They simplify an activity by decreasing the perceptual load until the final stages, and this becomes very interesting in the playing strategies of contact or evasion sports such as rugby or motorsport [12].

The aim of this systematic review is to discuss the important role of visual training and the consequent improvement in sports performance even when the visual system is impaired, and the need to use a multidisciplinary approach to achieve better results.

## 2. Materials and Methods

This review adhered to the guidelines of the preferred reporting items for reviews (PRISMA 2020).

### 2.1. Strategy

**Figure 1:**
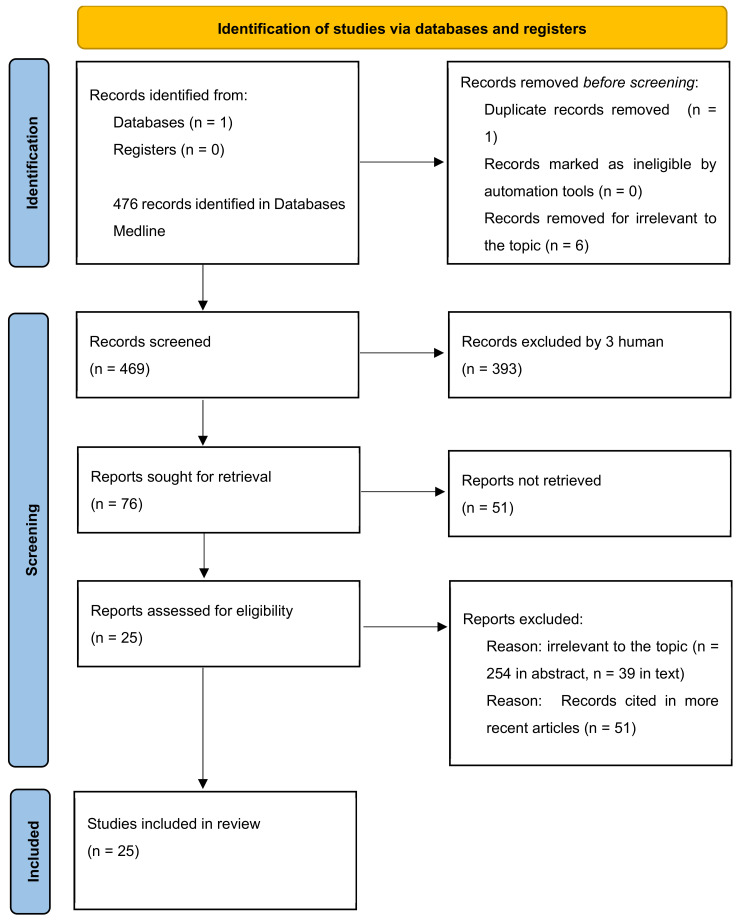
Identification studies.

In order to obtain controlled and randomized studies, observational studies, reviews and systematic reviews or meta-analyses on sport vision, we conducted searches in databases Medline (PubMed) starting in October 2022. Using the Mesh system research, the terms “Athletic Performance/education” [Mesh], “Athletic Performance/physiology” [Mesh], “Athletic Performance/statistics and numerical data” [Mesh], “Vision, Ocular” [Mesh], “Rehabilitation” [Mesh], “Sports” [Mesh], “Vision, Binocular/physiology” [Mesh], “PsychomotorPerformance/physiology” [Mesh], “Psychomotor Performance/physiopathology” [Mesh], “Pathology” [Mesh], “Saccades/physiology” [Mesh], “Visual Acuity” [Mesh], “Visual Cortex” [Mesh], “Vision Disorders” [Mesh], “Manipulation, Osteopathic” [Mesh], “Osteopathic Medicine” [Mesh] have been crossed with Boolean operators: AND, OR and NOT (Figure 1). MEDLINE was more likely to be consulted than any other information resource and is a critical clinical care tool that healthcare providers use to avoid adverse events, make changes to patient care, and answer clinical questions.

MeSH terms provide a great universal way to identify items. Official words or phrases labeled as MeSH terms represent a specific and described biomedical concept that particularly helps to identify a specific article for a topic and reduces the risk of bias [13,14].

- MESH: 4 Results

“Athletic Performance/education”[Mesh] OR “Athletic Performance/physiology”[Mesh] OR “Athletic Performance/statistics and numerical data”[Mesh]) and (“Vision, Ocular”[Mesh]) AND “Rehabilitation”[Mesh]

- MESH:13 Results

“Sports”[Mesh]) AND “Vision, Binocular/physiology”[Mesh]) AND “PsychomotorPerformance/physiology”[Mesh]) NOT “Psychomotor Performance/physiopathology”[Mesh]

- MESH:451 Results

“Sports”[Mesh]) AND “Vision, Ocular”[Mesh]) NOT “Pathology”[Mesh]

- MESH:451 Results

“Sports”[Mesh]) AND “Vision, Ocular”[Mesh]) NOT “Pathology”[Mesh]

- MESH: 5 Results

((((“Visual Acuity”[Mesh]) OR (“Visual Cortex”[Mesh] OR “Vision Disorders”[Mesh])) OR “Vision, Ocular”[Mesh]) AND (“Manipulation, Osteopathic”[Mesh] OR “Osteopathic Medicine”[Mesh])

The screening process was carried out by four authors: two orthoptists FM and IB and an osteopath AB and a psychologist MC.

### 2.2. Selection Criteria

We considered articles that were published in the English language between 1950 and 2023, time obtained to (“Sports”[Mesh]) AND ((“Vision, Ocular”[Mesh]) OR “Visual Acuity”[Mesh]). All of the published articles included in this collection were papers that reported on the role of sport vision in athletic performance and the development of visual function. Due to the variability in the age of the samples, the different techniques, the treatments among the participants in the studies and the finding of non-evaluable articles (two comments and an editorial), a meta-analysis was not conducted.

### 2.3. Evaluation Methods

The studies enrolled were assessed to understand the quality of the current literature, using, for clinical studies, the Jadad scale [15], for observational studies, the Newcastle–Ottawa scale [16], and, for reviews, the Amstar 2 scale [17] (Table 1).

The Jadad Scale was by far the most frequently cited and the most commonly used scale by the healthcare community. It has been adapted for use in many healthcare areas such as medicine, dentistry, psychology, and physical therapy. The Jadad Scale focuses only on randomization, blinding, withdrawals and dropouts to evaluate the methodological quality of primary research [15].

The Newcastle–Ottawa scale is one of the best-known scales for assessing quality and risk of bias in observational studies for several reasons. This tool is relatively quick to apply, although it requires the right amount of attention, and has good adaptability of its indices based on the topic investigated. Furthermore, it is validated for case-control and longitudinal studies [16].

The Amstar 2 scale is a major revision of the original AMSTAR tool, designed to evaluate systematic reviews that included randomized controlled trials, and it provides a broad assessment of quality, including flaws that may have arisen due to poor conduct of the review [17].

## 3. Results

Proprioceptive, visual, vestibular and cognitive systems continuously interact with each other and with the central nervous system, which integrates the information originating from them and balances them to ensure postural control under static and dynamic conditions [32]. However, each system makes its own contribution based on environmental conditions and the motor task performed [12,18].

### 3.1. Visual Skills in Different Sports

Generally speaking, the term stereopsis is used to define the ability to perceive depth or it is defined as the minimum distance at which it is possible to perceive depth. In strategy sports such as basketball, volleyball, and football, which involve actions of catching or intercepting the ball, specific visual skills (peripheral and spatial vision) are required as the actions depend on the position of the athlete, the action of the opponent and the position of the ball. It will mainly involve dynamic stereopsis to perceive the depth of an object or subject while following it. Sports such as golf, where the target is fixed, require static stereopsis (perception of the depth of a static object) [19]. Stereopsis is essential for the analysis of the environment in its three spatial dimensions, as well as for the evaluation of the speed and precision of movements in space and the distance between the athlete and the objects, as established by Fitt’s law, for which the time to reach an object or a given area depends on the distance of the object to be reached and the size of that object [20].

The greater the minimum distance at which depth can be perceived, the greater the stereopsis and perception of depth of the observed subject [4]. Stereopsis affects the time necessary to catch the ball: athletes with low stereopsis make more timing errors in catching rather than those who have a higher level, but there are not many differences regarding spatial errors. In addition, by increasing the speed at which the ball is pulled, it is possible to delay the beginning of the movement necessary to catch the ball increasing the success of the motor task [19,33].

The practice of sport-specific exercises (e.g., to catch a ball) does not provide the same results in subjects with low stereopsis as in those with good stereopsis, whose improvement in the activity of capture is more evident [34]. The difference between professional and amateur athletes should also be considered, as non-professional/amateur athletes use visual inputs as the main method for programming motor gestures; on the other hand, professional athletes integrate visual information with their sports experience; therefore, they encode the match (or the athletic performance) through a more complex visual integration system. Presta et al. [4] showed that professional athletes show better visual abilities than non-professional athletes [4]. It is also interesting to consider the place of training and rehabilitation of visual function [4].

### 3.2. Impact of Visual Training on Performance

A study conducted by Formenti et. al. [21] considered three working groups which were tested on the accuracy of specific volleyball skills and cognitive performance (clinical reaction time, executive control, perceptual speed) and then with sports training programs that required generic or specific volleyball motor actions in a no specific sport context, and a control group that performed a traditional volleyball training in a specific sports field [21].

After six weeks, the results showed that: “The accuracy of specific volleyball skills improved after a traditional volleyball training compared to vision training groups. In contrast, vision training groups improved in cognitive performance (reaction time, executive control, and perceptual speed), compared to the traditional volleyball training group”. This evidence suggests that the environment in which the exercises were performed plays a key role in the improvement of perception and action in sport-specific skills, supporting the ecological approach to sports learning [20,21]. It can be inferred that the training of the visual function in a non-specific sport context (with both generic and specific motor actions) improves cognitive performance, but it appears to be less effective for the improvement of specific sports skills. Stereopsis has a variable contribution in the various sports. The kind of sport, the role played, the setting, and the performance factors define the set of visual skills required and consequently, the relevance of stereopsis.

Athletes who practice a sport where it is required to grab and receive a ball have the significant ability to integrate visual and visual motor skills to control playing actions. In such sports, the athlete must follow the moving object and coordinate his action with the will to hit it or grab it and at the same time, evaluate speed and trajectory without losing sight of what is happening on the field. Although depth perception is not the only visual ability useful for the action of interception, it is nevertheless sufficient to discriminate athletes from non-athletes as they have a greater ability to integrate visual abilities and visual motor skills to control the action [4]. Studies conducted by Nascimento et al. showed that professional futsal players (5-a-side football) undergoing visual skills training distinguished themselves from the control group that did not play sports at competitive levels, only for an improvement in eye/hand coordination and a better reaction time (obtained after vision training, with better results on the field). Since, in the practice of futsal, peripheral vision is extremely relevant, it is recommended to use hand–eye coordination exercises to train and improve it [3]. To hit or catch an incoming ball you need to position yourself and move the object used for playing (hand/mash/racket) to the right place at the right time. There are three hypotheses on how this action is optimally carried out: according to some, athletes use predictive visual information about where the ball will be, according to others, it is used as an interaction between visual information and the movement required to keep the hand in the correct position, or finally, it is thought that the performance of this gesture is based on the use of previous knowledge to integrate inappropriate visual information. It is not possible to accurately estimate the absolute distance of the ball or its incoming speed, but the retinal images of a ball travelling at a constant speed contain monocular and binocular information about the direction of the ball, its depth and the arrival time [11].

Depth perception is possible thanks to binocular vision, the result of visual integrations from the right and left eye. Some evidence shows how the loss of vision of an eye could negatively affect the performance of a hitter, but it has emerged that a temporary visual occlusion is irrelevant. In sports that mainly use static stereopsis, such as golf, the sport vision training allows to increase in the probability of putting the ball in the hole and better evaluate the distance between them, as the athlete is able to focus more on proprioceptive and vestibular afferents than on visual signals, integrated into the programming of the swing gesture [19].

Do not forget the state of fatigue of the athlete that, in a proper assessment of performance related to visual function, should not be below anaerobic threshold; increases in lactate in the blood have been associated with a significant worsening of the mechanisms of attention [34,35].

Improved visual performance has been associated with a greater speed in transforming and decoding, at the cortical level, visual signals into motor actions by athletes [4,32].

### 3.3. How to Evaluate Visual Performance

The interest in sport vision is increasing and various methods are being sought to assess the athletes’ visual abilities. To improve communication between experts in sport vision and researchers, standardized test batteries have been developed and designed to assess different visual skills and maximize sports performance, and they are known as Pacific Sports Visual Performance Profile (PSVPP): a battery of 23 tests that assesses visual performance related to athletic competition [36]. This battery of tests provides a strict protocol related to instructions for the tests to be carried out in such a way as to be repeatable and provide quantitative information to experts of sport vision and researchers. Such tests highlight the visual skills associated with the motor task required. In particular, five areas of visual function are highlighted: visual sensitivity, accommodation/vergence, depth perception/eye teaming (coupling) central and peripheral visual recognition, and eye/hand/foot/body coordination [3]. Visual function plays a fundamental role in good sports performance [20] and it is a learned experience, not an automatic one [36], which accompanies the evolution of any athlete. Practically, all the athletes have an innate and a trainable part, so working on sport vision can make an athlete more and more competent in his sports practice and/or in his role within a team.

The improvement of visual abilities through oculomotricity exercises related to motor actions leads to a consequent performance improvement [2]. However, for optimal results, it is necessary to evaluate the specific visual performance required by the sport practiced and the role played, identify the athlete’s visual strengths and weaknesses and develop a specific sports vision training, and a specific monitoring and rehabilitation program.

## 4. Discussion

### 4.1. Visual Skills Training

The visual system is essential for the implementation of effective strategies for the productive regulation of dynamic stability during locomotion and optimization of strategies and adjustments to adapt to the travel path. Visual input is essential for proactive adjustments necessary to meet the different needs of travel route [11,12,22,23]

Taking into account the experience and the starting level of each athlete, it is possible to schedule a visual skills training plan for professional and amateur athletes. In sports where batting and receiving are the most important motor skills, training will focus on the speed and trajectory of the objects, integrating visual and visual motor signals with muscle training. In sports such as golf (in which you have to put the ball in the hole) or clay pigeon shooting, you will focus more on motor control through exercises that emphasize vestibular and proprioceptive signals. On the other hand, in strategy sports such as football or basketball, the exercises will activate the ‘stimulus–response’ system to improve peripheral vision, decision-making ability and speed of response time [4].

Among the exercises used to improve SV, there are specific orthotic exercises that use video or strobe images, or balance exercises with open and closed eyes [4]. With eyes closed, in fact, there is a reduction in strength in the lower extremities and different postural stability, but also a reduction in the tension of the masticatory muscles and the sternocleidomastoid muscle [4,24].

Abdollahipour et al. looked at the results of the use of visual information in association with an external attentional focus during a motor task involving body movements through space (body Projection) [25]. In particular, the subjects involved in the study were asked to make a high jump in a room with a 4 m high ceiling in perfect visual conditions or without the use of sight. Three tests were carried out: at first, there were no indications, in the second the subjects were asked to “focus on the ceiling and try to touch it” (external focus), in the third the participants were asked to focus on their fingers and try to bring them as high as possible (internal focus). The results indicated that regardless of visual conditions, subjects who maintain an external attentional focus jump higher than in other cases; in addition, the jump in subjects with full vision was significantly higher than subjects who performed the test in the absence of vision. Therefore, the study demonstrates the benefits of using an external attention focus in motor tasks that involve body movements, but also the independence between external attention focus and vision [26]. This information can also be used to gain a direct advantage on the court.

Burris et al. proposed a series of orthotic exercises to improve binocular vision of football, basketball and handball players, with positive effects on visual function after 8 weeks of training. In detail, after a preliminary eye warming, the training consisted of saccadic movements, eye movements of research, fixation, and exercises of dynamic vision, convergence, divergence, and eye relaxation exercises [20].

Clarl et al. used a different approach: they studied a vision training program as a prevention strategy against head trauma in sport using a specific training platform equipped with light signals [4]. There was an improvement in control and accuracy of fine eye movements, ocular convergence and fixation, just after the first six weeks of training, and a decay of these effects on stereopsis in athletes who interrupted the course. On the other hand, studies by Schoemann et al. report levels of stereopsis significantly high up to six months after stopping training. This result could be due to the adoption of a different method of measuring stereopsis: at first, the reaction time was evaluated at which the depth can be perceived (processing time); secondly, it was defined as the minimum distance at which the depth can be perceived [4].

### 4.2. The Role of Cognitive Factors

Standard rehabilitation aims at the recovery of static and dynamic postural stability through exercises for the stabilization of the trunk and the maintenance of the orthostatic position on unstable platforms such as proprioceptive pads or oscillating tablets, but it would also be useful to use what is called the “double task paradigm”, which consists of associated cognitive exercise while walking. To improve visual stability, however, the subject is asked to look at a stationary object during active movements of the head in the vertical and horizontal direction, or an attempt is made to stimulate the fixation sacs. In the case of subjects with neurological disorders, the use of technological devices, such as augmented reality, can help [18]. The athlete’s perceptual abilities are influenced by the visual reaction time in peripheral vision, and both factors are included in SV. Visual reaction time is related to information and cognitive processes that control and regulate movement, conditioned by the functions of the central nervous system and muscles. Motor reaction time is the time between the signal and the completion of an action; therefore, it has both sensory and motor characteristics [34,35].

The optimal central–peripheral simultaneity allows the player to absorb all the visual information about the object on which he focuses his vision and everything that is happening around him, without having to make any eye movement. For example, during a handball match, players collect information through vision while paying attention to different stimuli such as an unmarked teammate or a close opponent [2]. Within a team, coordination and reaction time turn out to be relevant aspects for optimal performance. By using the PSVPP test [36], Nascimiento et al. demonstrated that visual skills training will reduce reaction time, specifically eye–hand coordination, coordination, identification and reaction time (time anticipation, peripheral response, reaction time, visual memory) [3]. The speed of the reaction time, and consequently the speed of the motor response, depends on the type of training practiced by the athlete, his physical condition, his level of fatigue and his cognitive level (the smarter he is, the faster the reaction time) [3,32]. The development of visual and cognitive skills can benefit athletes in injury prevention [3].

“Vision is a learned experience, not an automatic one” [36]. The basics for processing visual information are learned in childhood, and the early use of the visual system is crucial in the development of visual skills required in athletics. In the human being, the visual system is not predominant but the ability to organize and perform a motor task is the result of the integration of auditory, visual, vestibular and proprioceptive abilities. The daily interaction of these abilities with the environment allows us to understand the environmental stimuli [3]. Coordination is driven by vision and task-oriented motor behavior with intention and emotion. The act of coordination between the primary eye–hand–mouth is the one that allows food to be brought to the mouth in order to satisfy a primary need for man: eating. Such an act is one of the most important ‘motor integrations’ and requires large-scale neural connections ‘on multiple cortexes’. The eye–hand–mouth coordination of the newborn is considered a precursor to subsequent self-feeding and mediated by the same network of cortical interconnections of the adult, although still underdeveloped. Futagi et al. [27] assumed that the act of touching the mouth and the early opening movement that the baby performs already in fetal life is connected with cortical maturation and preparation for feeding after birth [27]. As proof of this, the conclusions show that in the third trimester of pregnancy, the associated hand–mouth movements evolve from spontaneous and purposeless into motor behaviours that have the purpose of putting the hand in contact with the mouth. This means that the control of the upper brain mechanism becomes dominant on the neural circuit of the brainstem in view of birth [27]. The human sensorimotor system is governed by three main mechanisms: perception of external information, decision based on what is perceived, and execution/control of action based on what has been decided. The result is analyzed involving the sensory perception mechanism, the decision-making mechanism and the execution mechanism. Visually mediated actions are based on three systems: the gaze, to locate and fix the objects relevant to the task, the musculoskeletal system to perform the task and the visual system to provide information to the previous ones. These systems are supervised by a contour system that plans the sequence of actions and explains the activity performed at that time [3]. Each system has its own cortical representation, but is interconnected with the others. In the process of reaction time, the afferent pathway stimulus reaches the primary somatosensory cortex and the posterior parietal cortex, and this motor sense integration sends the information to area 6 of the motor cortex, where movement planning takes place. This moment of reaction time is known as pre-motor. In the motor period, information from area 6 is sent to area 4 of the motor cortex to generate the intention to initiate the movement, and the cerebellum plays an important role in guiding this future action [11]. Depending on the quality of cortical excitability, reaction time can be performed at a greater or lesser rate [3]. For all this, the vision must accompany the evolution of the athlete so that he can be increasingly competent in his integrated role in a team [3], but his optimal development is part of prevention for adult life. In fact, with age, the decay of all visual functions and physical performance increases exponentially, in particular high-contrast standard visual acuity and standard field of vision [28]. Poor visual performance in adulthood has been associated with poor physical performance. Spatial vision, visual field integrity, binocular vision or adaptation have significant relationships with functional results; indeed, they affect the daily functions of the elderly more than standard visual acuity, and one wonders whether working on these factors can reduce their disability.

No studies have been detected regarding the specific training of visual skills in the elderly, but Jobe et al. developed a trial called Advanced Cognitive Training for Independent and Vital Elderly (ACTIVE) that can be used as a model for future experimentation and to which this aspect can also be added [23]. The ACTIVE program aims to improve the cognitive and perceptual skills of elderly people at risk of functional decline and to postpone or prevent hospitalization by reducing health-related costs. The training is focused on memory, reasoning and speed of information processing, an aspect that refers to how much information in our perceptual field we are able to process at the cognitive level in a short exposure time (very useful when driving a vehicle) [22].

### 4.3. Osteopathy and Prevention

In the context of the development of visual function from a newborn to an adult, a ‘new healthcare profession’ [23,29,37,38], osteopathy, should be considered. It is likely that osteopathy will be framed within the area of prevention so as not to overlap skills with fellow rehabilitators. The osteopathic treatment (OMT) has the main purpose of researching and solving the somatic dysfunction (SD), considered as a key element of osteopathic practice. SD is listed in the current version of the International Classification of Hospital Diseases (ICD) [30]. It was used to be considered a milestone of the first regional osteopathic anatomical approaches to the person, but now it has come to be seen as a deterioration in the activity of the body’s regulatory function related to the body structure, to a region or a generalized body pattern who involve the entire organism [37]. Therefore, it is essential to prevent its progression through appropriate interventions from an early age. Manual therapy is a non-invasive method, and in the literature, it is possible to find applications of the osteopathic manipulative treatment from the pre-term newborn to the elderly with a positive impact on health and quality of life [23,31,38]. Many conditions can be helped by osteopathic manipulative treatment. These conditions include headache; orofacial pain; cranial nerve entrapment; vertigo; tinnitus; closed head trauma and concussion; temporomandibular joint dysfunction; ocular dysfunction, such as strabismus and astigmatism; various otolaryngological disorders, such as Eustachian tube dysfunction, otitis media and chronic pharyngitis; dental pain and malocclusion; and plagiocephaly and feeding difficulties in children [31,39]. Osteopathic scientific literature on visual functions is poor [38] but there is great potential for future studies of OMT on the visual path since it is linked to most of the central nervous system and parts of the peripheral nervous system [15]. Osteopathic collaboration is also desirable in the field of cognitive neuroscience, as the study by Akkas et al. [40] examines the effect of the OMT on anaerobic performance and lactate clearance in male athletes and shows that the concentration of lactate in the blood was significantly lower in the OMT group compared to the control group at 15 and 30 min (*p* < 0.05), suggesting that OMT can improve lactate elimination without affecting athletes anaerobic performance [40]. Both professionals and amateurs will achieve improvements through this type of approach, but it is important to take into account the level of experience of each athlete and to start with the basics for novices.

### Limitations and Risk of Bias of the Study

The limitation of this review is the uneven sample size of the recruited studies, which did not allow for a meta-analysis. The literature survey of only one database could represent a limitation for potentially eligible studies. The studies analysed often have a sample number that is not statistically representative and without a control group and above all there is not a standardized test used to measure performance, PSVPP is mentioned only once [3].

## 5. Conclusions

The panorama of the studies investigated by this review confirms the importance of the SV to improve sports performance and reduce injuries, and from 1950 to the present, an increasing interest has been devoted to SV. In the literature, there is a close collaboration between SV and sports performance, and the results are directly correlated with the specific exercise of vision [3,12,21]. The interest in sports vision has led researchers to investigate an evaluation method. To date, the most effective is the PSVPP [3,36].

The cognitive processes that control and regulate movement, conditioned by the functions of the central nervous system and muscles, are equally important. An athlete with good cognitive systems, good reaction times and good peripheral vision is able to grasp information from game actions and improve his performance [34,35].

It remains of undoubted relevance of the orthoptic with regard to the rehabilitation of the oculomotor apparatus, in particular for the problems of visual function related to amblyopia and stereopsis, while offering collaborative ideas with the fellow rehabilitation of physiotherapy in the postural and motor responsibility of the athlete. Although the studies noted are still insufficient, the figure of the osteopath also seems to be useful, preventive and complementary to support visual rehabilitation.

A fruitful collaboration with sports psychologists and cognitive neuroscience is plausible, which are still all in ‘power’ and far away from the collaboration with the osteopath [29,30] who, although present in the clinical field, does not have their roots in the literature, and this offers a broad scenario for further studies.

## Figures and Tables

**Table 1:** Evaluation of enrolled studies.

Author/Year	Reference	Type of Study	Rating Scale	Scale Score	Aim of the Study	Population Studied	Sport Studied	Parameters Analyzed	Means of Evaluation
Nascimento 2020	[2]	Comment	Not applicable	Not applicable	Use Sport Vision citation networks in order to analyze the relationships between different sports	Not applicable	Not applicable	Not applicable	Not applicable
Nascimento 2021	[3]	Cross-sectional Study	Newcastle–Ottawa scale	*******	Explore if a group of futsal players, in a laboratory setting, would have better results in actions in which vision is relevant than a control group that has nothing in common with futsal or any other sport.	10 futsal athletes 35 control group	Futsal	COI-SV software (https://coi-sl.es/software/ accessed on 9 may 2024)	Tests that measure the most relevant visual abilities for futsal
Presta 2021	[4]	Review	Amstar 2	>1	The purpose of this review is to take stock of the effects of stereopsis on athletic performance, examining, at the same time, training tools to improve visual abilities and potential differences in the visuomotor integration processes of professional and non-professional athletes.	Professional and non-professional athletes	Interceptive sports (baseball, cricket and tennis,). Strategic Sports (basketball, soccer and volleyball)	Dynamic stereopsis, visual skills (peripheral and spatial vision)	Nike SPARQ—Speed, Power, Agility, Reaction, and Quickness—Sensory Training Station
Sharma 2018	[5]	Review	Amstar 2 scale	>1	Review of clinical studies on the restoration of alignment and stereopsis in cases of amblyopia, esotropia, strabismus. Medical treatment combined with exercises/video games in monocular and binocular vision.	Children and adults	Not applicable	Stereopsis in children and in adults.	Medical treatment and perceptual training with monocular or binocular video games
Matthis 2018	[6]	Cohort Study	Newcastle–Ottawa scale	**	Examine the relationship between vision and walking	Walkers	Trekking	Positive Science mobile eye tracker Motion Shadow inertial measurement unit (IMU)	Techniques that record the gaze of the subjects and the kinematics of the body during the journey
Bonnen 2021	[7]	Observational Study	Newcastle–Ottawa scale	**	Examine the role of binocular information in the visuomotor control of walking on complex ground	8-normally-sighted participants 2 had anisometropic amblyopia 2 participants had strabismus	Trekking	Battery of acuity, stereoacuity, and motor tests.	Evaluation of visual tests and measurements of physical function.
Buckley 2010	[8]	Comparative Study (controlled clinical study)	Jadad scale	2	Adaptation of the gait in individuals with long-standing stereopsis deficit compared to subjects with normal vision and partial blurring of vision (condition caused for the test).	12 visually normal subjects and 16 individuals with deficient stereopsis due to amblyopia and/or its associated conditions	Obstacle-crossing performance. Trial with floor-based obstacles of different heights (7–22 cm).	Subjective refraction and binocular vision assessment (cover test, ocular motility, stereo threshold).	Test Chart 2000, Thomson Software (https://thomson-software-solutions.com/products/test-chart-solutions/ accessed on 9 may 2024) Solutions, Herts UK.
Timmis 2012	[9]	Comparative Study (controlled clinical study)	Jadad scale	1	Study how patients with central visual field loss (CFL) complete adaptive gait tasks compared to subjects with normal vision and determine if the difficulty of the task significantly affects the control of the movement.	Ten patients with CFL and 12 visual normals	Walking through floor-based obstacles (of different heights, 5 and 10 cm) and also across an unobstructed laboratory (no obstacle present)	Motion analysis systems; Humphrey Field Analyzer.	Analysis of the kinematics of human movement for each task.
Zhao 2021	[10]	Cohort Study	Newcastle–Ottawa scale	****	The role of binocular vision in virtual locomotion	Ten people (7 males, 3 females, age: 24–39	Walking	Stride length, Stride height, Foot lifting distance to obstacles or gaps, Foot planting distance to obstacles or gaps, Foot clearance to obstacles, Foot speed of mid swing, Number of strides, Number of collisions.	Walking experiments in virtual environments using a linear treadmill and a novel projected display known as the Wide Immersive Stereo Environment (WISE)
Cinelli 2008	[11]	Analytical study	Newcastle–Ottawa scale	***	Strategies used to pass through a swinging target and analysis of the movement to reach it.	7 healthy female students (age: 20–24 years)	Not applicable	Kinematic data	Optotrak (Northern Digital Inc., Waterloo, ON, Canada) system.
Mazyn 2007	[12]	Non-randomized controlled study	Jadad scale	3	The contribution of stereopsis in the interceptional task.	23 female, (22.2 +/− 4.4 years of age)	Tennis	Number of interceptions of balls in training with sessions of different speeds and	Each trial was scored as a catch or amiss.
Manzari 2022	[18]	Editorial	Not applicable	Not applicable	Evaluate the gaze and the postural function both in static and dynamic conditions; new rehabilitation strategies for balance disorders.	Not applicable	Not applicable	Video Head Impulse Test	The gaze stability exercises, standard rehabilitation, galvanic vestibular stimulation
Dalton 2015	[19]	Cohort Study	Newcastle–Ottawa scale	**	Assess ocular dominance both in primary gaze and direct gaze.	31 golfers (14 amateur, 7 club professionals, and 10 top professionals)	Golf	Measuring pointing ocular dominance	Retrospective analysis of data collected during optometric examinations, optometric screenings, and putting vision assessments
Burris 2018	[20]	Cohort Study	Newcastle–Ottawa scale	****	Evaluate connections between sensorimotor skills and sports performance on the field	252 professional baseball players	Baseball	Sensorimotor abilities	Nike Sensory Station assessment battery
Formenti 2019	[21]	Randomized Controlled Trial	Jadad scale	3	Hypothesis of the study: “Does visual training in a non-specific context improve sport-specific skills?”	51 volleyball players	Volleyball	Volleyball-specific skills (setting, serving, passing), clinical reaction time, visual search task.	Sport vision training programmes
Regan 1997	[22]	Review	Amstar 2	>1	Visual factors in hitting and catching	Not applicable	Cricket	Not applicable	Application of sports physics
Guzzetta 2009	[23]	Clinical trial	Jadad scale	2	To explore the effects of body massage in preterm infants and found that massage accelerates the maturation of electroencephalographic activity and of visual function, in particular visual acuity.	Rat pups	Not applicable	Level of IGF-1 in the cortex	Body massage
Zieliński 2022	[24]	Review	Amstar 2 scale	>1	Connections between the organ of vision and the stomatognathic system	Not applicable	Not applicable	Not applicable	Not applicable
Abdollahipour 2016	[25]	Observational Study	Newcastle–Ottawa scale	**	The influence of vision on attentional focus during the execution of movements.	24 adults, average age 25 years.	Maximum vertical jump	Measurement of jump height	Jump with external focus, internal focus, and control (no-focus instruction).
West 2002	[26]	Cross-sectional study	Newcastle–Ottawa scale	*******	Evaluate the relationship between visual functions and physical performance in older adults.	782 adults aged 55 and older	Walking and balance	Comprehensive battery of vision tests and physical function measures.	Evaluation of visual tests and measurements of physical function.
Futagi 2017	[27]	Review	Amstar 2 scale	>1	Clarify the ontogenetic meaning of coordination during the first months of life and its neuronal mechanism according to the current knowledge of cognitive neuroscience	n 117 full-term normal newborns,	Not applicable	Hand-mouth coordination, Eye-hand coordination, Eye-mouth coordination	Clinical evaluation of video footage.
Jobe 2001	[28]	Control Clin Trials	Jadad scale	5	Cognitive training and its effects on daily functioning	2832 participants (predominantly female and ranging in age from 65–94 years with an average age of 73.6 years)	Not applicable	Tests of memory, telephone interview, in-person interview, MOS 36-item Short Form (SF-36)	Tests of memory, reasoning, and speed of information processing
Bach 2017	[29]	Comment	Not applicable	Not applicable	With the numerous limitations that the authors discussed, including some of those we have mentioned, we question the validity of this study’s results (Sandhouse 2016).	Not applicable	Not applicable	Not applicable	Not applicable
Sandhouse 2016	[30]	Randomized Controlled Trial	Jadad scale	4	The authors conducted a study that examined whether osteopathic cranial manipulative medicine produced a measurable change in visual function in adults with cranial asymmetry.	Adult volunteers aged between 18 and 35 years with unremarkable systemic or ocular history were recruited. 47 in the treatment group and 42 in the sham therapy group.	Not applicable	Preintervention and postintervention ophthalmic examinations consisted of distance visual acuity testing, accommodative system testing, local stereoacuity testing, pupillary size measurements, and vergence system testing.	Osteopathic cranial manipulative medicine
Mardini 2019	[31]	Description of techniques	Not applicable	Not applicable	Many conditions may be helped by osteopathic manipulative treatment. These conditions include ocular dysfunction, such as strabismus and astigmatism. Description of the frontal and parietal lifting techniques	Not applicable	Not applicable	This video was produced by Touro University College of Osteopathic Medicine-CA.	Osteopathic Cranial Manipulative Medicine: Frontal and Parietal Lift Techniques

scores in * ≥ 7–9, 4–6, <4 are considered low, intermediate, and high risk, respectively.

## Data Availability

Not applicable.
